# Reliability of Ayurvedic Diagnosis for Knee Osteoarthritis Patients: A Nested Diagnostic Study Within a Randomized Controlled Trial

**DOI:** 10.1089/acm.2018.0273

**Published:** 2019-09-12

**Authors:** Christian S. Kessler, Antonio Morandi, Abhimanyu Kumar, Kartar S. Dhiman, Shivenarain Gupta, Katja Icke, Carina Bühner, Elmar Stapelfeldt, Manfred Wischnewsky, Ludwig Kronpaß, Vijayendra Murthy, Andreas Michalsen, Claudia M. Witt

**Affiliations:** ^1^ Charité – Universitätsmedizin Berlin, Corporate Member of Freie Universität Berlin, Humboldt-Universität zu Berlin, and Berlin Institute of Health, Institute for Social Medicine, Epidemiology and Health Economics, Berlin, Germany.; ^2^ Department for Complementary Medicine, Immanuel Hospital Berlin, Berlin, Germany.; ^3^ Ayurvedic Point, School of Ayurvedic Medicine, Milan, Italy.; ^4^ Central Council for Research in Ayurvedic Sciences (CCRAS), New Delhi, India.; ^5^ All India Institute of Ayurveda, New Delhi, India.; ^6^ European Academy of Ayurveda, Birstein, Germany.; ^7^ Department of Kaya Cikitsa, J.S. Ayurveda College & P.D. Patel Ayurveda Hospital, Nadiad, India.; ^8^ Department of Mathematics and Computer Science, University of Bremen, Bremen, Germany.; ^9^ AyurSan-Zentrum Sankt Salvator, Bad Griesbach, Germany.; ^10^ Primary Care and Population Sciences, Faculty of Medicine, University of Southampton, Southampton, United Kingdom.; ^11^ Institute of Complementary and Integrative Medicine, University Hospital Zurich, University of Zurich, Zurich, Switzerland.; ^12^ Center for Integrative Medicine, University of Maryland School of Medicine, Baltimore, Maryland.

**Keywords:** Ayurveda, Ayurvedic, diagnostic, reliability, osteoarthritis, whole systems of medicine, complementary medicine

## Abstract

***Background:*** Ayurveda is a traditional Indian system of medicine. The customized Ayurvedic approach consists of a combination of several diagnostic procedures and subsequent individualized therapeutic interventions. Evaluation of inter-rater reliability (IRR) of Ayurvedic diagnoses has rarely been performed. The aim of this study was to evaluate IRR of Ayurvedic diagnosis for patients with knee osteoarthritis.

***Methods:*** A diagnostic reliability study of 30 patients and 4 Ayurvedic experts was nested in a randomized controlled trial. Patients were diagnosed in a sequential order by all experts utilizing a semistructured patient history form. A nominal group technique as consensus procedure was performed to reach agreement on the items to be diagnosed. An IRR analysis using Fleiss' and Cohen's kappa statistics was performed to determine a chance-corrected measure of agreement among raters.

***Results:*** One hundred and twenty different ratings and 30 consensus ratings were performed and analyzed. While high percentages of agreement for main diagnostic entities and the final Ayurveda diagnosis (95% consensus agreement on main diagnosis) could be observed, this was not reflected in the corresponding kappa values, which largely yielded fair-to-poor inter-rater agreement kappas for central diagnostic aspects such as *prakriti* and *agni (*κ values between 0 and 0.4). Notably, agreement on disease-related entities was better than that on constitutional entities.

***Conclusions:*** This is the first diagnostic study embedded in a clinical trial on patients with knee osteoarthritis utilizing a multimodality whole systems approach. Results showed a contrast between the high agreement of the consented final diagnosis and disagreement on certain diagnostic details. Future diagnostic studies should have larger sample sizes and a methodology more tailored to the specificities of traditional whole systems of medicine. Equal emphasis will need to be placed on all core diagnostic components of Ayurveda, both constitutional and disease specific, using detailed structured history taking forms.

## Background

Ayurveda is the most common traditional system of medicine of India and has a significant impact on the health economy.^1–3^ Its popularity is recently increasing also in the West due to its person-centered approaches.^4–7^ Its main concern is the maintenance of an abiding health, preventing as well as treating diseases. Health is a state derived from the dynamic balanced interplay of several constituents: physiologic, mental, social, and spiritual.^4,8–13–15^ The overall emphasis on attaining health and overcoming disease in Ayurveda can be encapsulated as the concept of resilience, the capacity of a system to continually change and adapt to external stimuli and variations while remaining within critical thresholds.

The factors determining resilience in an individual are conceived in Ayurveda as relative balance/imbalance between the functional principles called *dosha*, which are as follows: *vata*, *pitta*, and *kapha*. *Vata dosha* is related to the concept of movement/kinetics, *pitta dosha* to the concept of transformation/metabolism, and *kapha dosha* to the concept of cohesion/anabolism^16^ (Supplementary Data S1). Additional essential paradigmatic elements are *agni*, the digestive and metabolic principle, *mala*, the physiologic waste products, *ama*, the result of dysmetabolism and digestive errors along with defective transformation of the body tissues termed *dhatu.*^17^

**FIG. 1.**
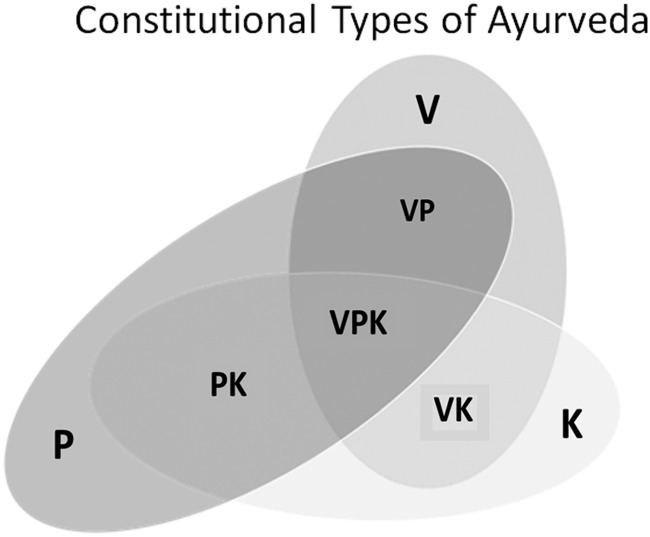
Constitutional types of Ayurveda. K, *kapha*; P, *pitta*; VK, *vata-kapha*; V, *vata*; VP, *vata-pitta*; VPK, *vata-pitta-kapha.*

These factors contribute to a person's psychophysical constitution called *prakriti*. The variations of pathologic manifestations of disease conditions as well as the diverse individual responses to therapies are deemed to be the result of the differences in *prakriti*. The *prakriti* of individuals are classified as one of the seven types, based on *vata*, *pitta*, *kapha,* and their combinations: *vata-pitta*, *vata-kapha*, *pitta-kapha,* and *vata-pitta-kapha*^14,15,18^ (Fig. 1).

The assessment of *prakriti* can provide information about a person's predisposition to disease/s and clues for primary prevention, as confirmed by recent studies.^6,19–25^

### Diagnostic considerations in Ayurveda

Evidently, the diagnostic process in Ayurveda requires different criteria from those of conventional medicine. In brief, it is composed of the assessment of *prakriti*, the inborn constitution, and the assessment of the imbalance, *vikriti*, which is identified with the diseased “*milieu interieur*.” *Prakriti* helps identify possible directions a disease development may take.^26,27^ The assessment of *vikriti* serves to define the variables of the pathologic changes on structural and functional levels of the patient. Other factors, such as social and climatic determinants, are also taken into consideration while establishing a diagnosis.^28–30^

The therapy, personalized on the basis of all these before mentioned factors, will then be adjusted according to the progression of the disease. The diagnostic process leads to both identification of treatment/therapy and its modifications. Therefore, the diagnostic process is characterized by high complexity with multidimensional variables, the therapy not only being targeted to the disease but being tailored to the condition of the individual patient, including Ayurveda-specific etiologic and pathogenetic (*hetu*, *samprapti*) concepts.^26,31^

Thus, it is impossible to generate a precise equation to perfectly translate modern medical nomenclature one to one with Ayurveda's. However, in some cases it is possible to identify sufficient correspondences to reach a reasonably good comparison.^32^ For instance the condition of osteoarthritis can be well correlated to the Ayurvedic diagnosis of *sandhi-gata-vata*, which is very similar to osteoarthritis in both etiopathogenesis as well as its clinical manifestation. According to classic descriptions this is caused by a migration of aggravated *vata* to the *sandhi* (joint structures).^33^ As in modern medicine, a proper diagnosis is the basis for planning of an Ayurvedic treatment.^34^

Besides confirming the diagnosis as *sandhi-gata-vata* there are some additional diagnostic components necessary for developing a treatment plan. What is the mode of *vata* aggravation leading to a decay of body tissues or a blockage in the pathways of *vata*? Is there an association of any other *dosha*, for example, *pitta* or *kapha* or both? Which specific properties of these *doshas* are key players in the etiopathogenesis? Although the examination of constitutional aspects, for example, *prakriti*, *agni*, etc., is also emphasized in Ayurvedic texts and practice, these are often difficult to assess accurately due to an overshadowing of constitutional features by disease characteristics. A proper constitutional assessment is possible only if the patient is in good acquaintance with the clinician; otherwise, the physician must rely on the patient's own descriptions about his/her constitutional features.

This approach generates some problems when designing a clinical study according to the rules of randomized controlled trials (RCTs). While the applicability of RCTs to Ayurveda is still emerging, to conduct reliable studies comparable with and acceptable by the standards of biomedicine, it is essential to construct innovative study designs that recognize the effectiveness of Ayurveda and utilize the multidimensional complexity of the Ayurvedic diagnostic as well as its therapeutic approaches.^5,7,35–37^ Recently, a novel design of clinical trial that considers the complexity of Ayurveda has been developed and proposed.^5,7^ However, the reliability of the complex processes of Ayurvedic diagnosis in the context of clinical trials still has not been widely investigated.

It is widely acknowledged that inter-rater reliability (IRR) for diagnostic methods is a critical component that is required to recognize the validity of data in a study and its interpretation. According to classical test theory (CTT), reliability is defined as the ratio of true score variance to the observed score variance and is represented by reliability coefficients. In CTT, the observed score X is a composite of the true score T and the error E (X = T+E).^38^ Thus, reliability coefficient kappa is used to estimate the amount of measurement error in assessments and is generally expressed as a coefficient ranging from −1 to 1.

Low reliability means that the error component is large for that assessment. For example, an IRR estimate of 0.30 would indicate that 30% of the observed variance is due to true score variance or similarity in ratings between coders, and 70% is due to error variance or differences in ratings between coders. Although higher reliability is always preferable, there is no fixed threshold to discriminate “reliable” from “unreliable” scores.^39^ Reliability coefficients may be low even though there are high levels of agreement and even though individual ratings are accurate.^40^ Whether a given value implies a good or a bad rating system or diagnostic method depends on what model one assumes about the decision making of raters.^41^ Kappa is influenced by trait prevalence (distribution) and base rates.^42^

Reliability is one of eight criteria for evaluating the patient-based outcome for any specific clinical trial: appropriateness, reliability, validity, responsiveness, precision, interpretability, acceptability, and feasibility.^43^ There are several types of reliability estimates. IRR or “reproducibility” denotes in clinical settings the extent to which physicians agree with each other in their diagnosis and treatment. In clinical studies where clinical diagnosis relies upon the personal skills of physicians, IRR is often perceived as the standard measure of research quality. High levels of disagreement among raters suggest weaknesses in the underlying qualitative notions and the diagnostic methods, including the possibility of poor operational definitions, categories, and perhaps deficiencies in the inter-rater training.

Inter-rater agreement (IRA) emphasizes the interchangeability or the absolute consensus between raters.^44^ It measures the extent to which different raters assign the same precise value for each item being observed. IRA is the “best” method to use when many ratees receive the same rating.^44^

Intra-rater reliability also known as “test-retest reliability” or “repeatability” is used to assess the consistency of the same rater at different times for the same group of patients.

While a large number of studies exist for IRR of conventional diagnostic methods, a comparatively small portion of such studies exists for the assessment of IRR in traditional systems of medicine, with best data for Chinese medicine.^45,46^ Few comparable studies have also been performed for Japanese traditional systems of medicine and traditional Indian medicine Ayurveda.^47–49^ Even though all above-mentioned traditional systems of medicine assessed share the centrality of the individual constitution, they use quite different toolboxes for performing diagnostics. Only one previous study has tried to comprehensively investigate IRR of the pulse, tongue, and constitution (*prakriti*) used in Ayurveda,^47–49^ demonstrating according to the Landis and Koch (LK) scale^39^ poor-to-moderate levels of IRR.^50^

Overall, diagnostic reliability studies in Ayurveda are thus obviously still in a pioneer stage looking at the available data. The aim of this study was to evaluate IRR of an Ayurveda diagnosis for patients with osteoarthritis of the knee.

## Materials and Methods

### Design overview

A diagnostic reliability study with 30 patients with knee osteoarthritis and 4 Ayurveda expert physicians (P1–P4) was nested into a RCT^5,7^ (see Supplementary Fig. S1). The first 30 patients who were found eligible for participation in the main RCT during the screening process were included; no additional inclusion or exclusion criteria other than those described in the RCT methodology were applied.^5,7^

The study took place on four sequential days from November 15 to 18, 2010 in a German university hospital setting in Berlin. The 30 patients of the RCT were initially diagnosed by the study physician, expert in Ayurveda. These patients were then subjected to sequential sessions (max. 40 min duration) of Ayurvedic diagnostics performed by three more independent experts in Ayurveda. The team of experts consisted of one Indian physician who had undergone regular university education for Ayurveda in India (B.A.M.S. with 5.5 years of training plus 3 years postgraduate Ayurveda training) and conventionally educated European physicians with additional Ayurveda training (≥500 h of academic training in Ayurveda plus ≥2 years of continuous clinical experience with Ayurveda in Europe). All experts were particularly trained in the Ayurvedic discipline of *kaya-cikitsa* (best translated as “general medicine”), where the most common Ayurvedic diagnostic equivalents of osteoarthritis are described in a detailed way.

All Western experts had a similar conventional experience level of knee osteoarthritis comparable with conventional general practitioners. The diagnostic procedures took place in three different rooms of the same building. The setting ensured that the experts could not communicate with each other or exchange patient information. Rooms, equipment, atmosphere settings, and overall working conditions were comparable in all rooms. Each patient could be registered and allocated only once for this purpose. The patients were strictly instructed not to share any information with the other experts or any other study personnel while moving on to the next diagnostic session.

The main RCT had an open-label design, and prediagnosed knee osteoarthritis was the main inclusion criterion and Western “target condition.” Therefore, all trial physicians, including all those physicians involved in the diagnostic study, were *a priori* aware of the conventional diagnosis “osteoarthritis of the knee” of all participants.^5,7^

All experts had to use the same semistructured, trilingual (German, English, and Sanskrit) patient history form (Supplementary Data S2) to document their diagnostic findings. At the end of each of the 4 days, consensus meetings including the center's trial physician and the three Ayurveda experts for the diagnostic validation study were conducted, allowing a maximum of 10 min discussion time for each patient's data.

The nominal group technique^51^ was used to reach final agreements on Ayurveda diagnoses and therapeutic recommendations for each case, which were documented in a consensus form (see Supplementary Data S3). From the 30 patients, 9 were seen on days 1–3 each and 3 patients on day 4. A total of 150 ratings (150 = 30 × (4 + 1(consensus)) were performed. A facilitator (a physician not directly involved in the diagnostic study) was also present, providing indirect or unobtrusive assistance, guidance, and supervision.

The study was part of a RCT^5,7^, registered at clinicaltrials.gov under NCT01225133 (initial release 10/06/2010). It followed the Declaration of Helsinki and Good Clinical Practice guidelines for trial conduct. Participants provided written informed consent before taking part and were not reimbursed for participation.

### Statistics

An IRR analysis using Fleiss' kappa statistic (or Cohen's kappa statistic in the case of pairwise IRR or intraobservability) was performed for the assessment of agreement among raters.^39,50^ Two possible uses of kappa were distinguished: as a way to test rater independence (i.e., as a test statistic) and as a way to quantify the level of agreement. The first use involves testing the null hypothesis^39^ that there is no more agreement that might occur by chance given random guessing. The second use of kappa is to quantify actual levels of agreement. In this case, kappa's calculation uses a term called the proportion of expected agreement. This term is interpreted as the proportion of times raters would agree by chance alone.

Overall interpretations of the kappa statistic were based on the criteria described by LK. Possible values for kappa statistics range from −1 to 1, with 1 indicating perfect agreement, 0 indicating completely random agreement, and −1 indicating “perfect” disagreement. Kappa is positive when the agreement exceeds what is expected by chance.

The level of reliability is defined as follows: κ values of 0.81–0.99 were considered to represent almost a perfect reliability; 0.61 to 0.80—substantial reliability; 0.41 to 0.60—moderate reliability; 0.21 to 0.40—fair reliability; and 0.01 to 0.20—slight reliability and values ≤0 as indicating no agreement.^39^ Multiple response analysis was used when a question could be answered multiple valid times. A *p*-value of <0.05 was taken as statistically significant. Statistical analysis was performed using IBM SPSS 22 and R version 3.2.

## Results

All 30 patients received 4 Ayurvedic diagnostic sessions and a consensus discussion, resulting in a total of 150 different ratings.

### IRR of *prakriti* assessment

The level of pairwise reliability between the four physicians' (P1–P4) assessments is shown in Table 1. The IRR was slight (κ = 0.02–0.13). None of the pairwise kappas were moderate, substantial, or perfect (details shown in Table 1). As a consequence of the low pairwise reliability, the overall agreement among the four raters is also slight but not significant (Fleiss' κ = 0.02; 95% confidence interval [CI, −0.07 to 0.12]; *p* = 0.607). Looking category-wise at the levels of agreement for *prakriti*, a significant positive value (κ = 0.22; 95% CI [0.08–0.37]; *p* = 0.003) was obtained only for PK (*pitta-kapha*). All other values of kappa were negative; that is, the levels of agreement were poor according to the LK categories. When restricting the analysis to the three Western physicians, a poor nonsignificant level of agreement (Fleiss' κ = −0.03; 95% CI [−0.16 to 0.10]; *p* = 0.654) was also obtained.

**Table 1.** Levels of Reliability for *Prakriti* and *Agni* Assessment: Pairwise Kappas

	*P1*	*P2*	*P3*	*P4*
*Prakriti*				
P1	1	0.12 (*p* = 0.265)	0.02 (*p* = 0.675)	0.10 (*p* = 0.060)
P2	0.12 (*p* = 0.265)	1	0.10 (*p* = 0.134)	0.13 (*p* = 0.050)
P3	0.02 (*p* = 0.675)	0.10 (*p* = 0.134)	1	0.12 (*p* = 0.303)
P4	0.10 (*p* = 0.060)	0.13 (*p* = 0.050)	0.12 (*p* = 0.303)	1
*Agni (prakriti)*				
P1	1	0.27 (*p* = 0.034)	0.29 (*p* = 0.009)	0.16 (*p* = 0.055)
P2	0.27 (*p* = 0.034)	1	0.33 (*p* = 0.003)	0.11 (*p* = 0.222)
P3	0.29 (*p* = 0.009)	0.33 (*p* = 0.003)	1	0.10 (*p* = 0.191)
P4	0.16 (*p* = 0.055)	0.11 (*p* = 0.222)	0.10 (*p* = 0.191)	1
*Agni (vikriti)*				
P1	1	0.00 (*p* = 0.988)	0.24 (*p* = 0.044)	0.05 (*p* = 0.597)
P2	0.00 (*p* = 0.988)	1	0.37 (*p* = 0.002)	0.43 (*p* < 0.001)
P3	0.24 (*p* = 0.044)	0.37 (*p* = 0.002)	1	0.33 (*p* < 0.001)
P4	0.05 (*p* = 0.597)	0.43 (*p* < 0.001)	0.33 (*p* < 0.001)	1

P1–4, physician 1–4; *prakriti*, individual constitution; *agni (prakriti)*, constitutional digestive capacity as assessed by interrogation; *agni (vikriti)*, pathophysiologic assessment of digestive capacity.

### Inter-rater reliability of *agni (prakriti)* assessment

The pairwise IRR for *agni (prakriti)* as shown in Table 1 is slight or fair (κ = 0.10–0.33). The overall agreement among the four raters is slight (Fleiss' κ = 0.20; 95% CI [0.10–0.29]; *p* < 0.001). When looking category-wise at the levels of agreement for Ayurvedic subcategories of *agni (prakriti)*, statistically significant fair reliability (κ = 0.24 resp. 0.30; *p* < 0.001) was obtained for *sama-* and *vishama-agni*. For *manda-* and *tikshna-agni,* a significant agreement (*p* > 0.05) could not be found. When restricting the analysis again to the three Western physicians, a statistically significant fair agreement (Fleiss' κ = 0.29; 95% CI [0.15–0.43]; *p* < 0.001) was obtained.

### Inter-rater reliability of *agni (vikriti)* assessment

The pairwise inter-rater reliabilities for *agni (vikriti)* as shown in Table 1 are between no agreement (κ = 0.002, *p* = 0.988) and statistically significant moderate reliability (κ = 0.43, *p* < 0.001). The overall agreement among the four raters is slight (Fleiss' κ = 0.22; *p* < 0.001). When looking category-wise at the levels of agreement for *agni (vikriti)*, statistically significant substantial reliability (κ = 0.72; *p* < 0.001) and statistically significant moderate reliability (κ = 0.43; *p* < 0.001) were obtained for *sama agni* and *vishama agni*, respectively. For *sama agni* and *manda agni,* an agreement could not be found at all except by chance given random guessing (*p* > 0.05).

The IRR of the various practitioners with the consensus agreement showed the following results: P1 (κ = 0.11; *p* = 0.368), P2 (κ = 0.68; *p* < 0.001), P3 (κ = 0.45, *p* < 0.001), and P4 (κ = 0.63; *p* < 0.001).

### Intra-rater reliability of *agni (prakriti)* versus *agni (vikriti)* assessment

The results for intra-rater reliability (test–retest reliability) of *agni (prakriti)* (constitutional digestive capacity as assessed by interrogation) versus *agni (vikriti)* (pathophysiologic assessment of the digestive capacity) to assess the consistency of the same rater at different times for the same group of patients are substantial to almost perfect: P1 (κ = 0.61; *p* < 0.001), P2 (κ = 0.83; *p* < 0.001), P3 (κ = 0.83; *p* < 0.001), and P4 (κ = 0.83; *p* < 0.001). All results are highly significant.

### Fleiss' kappa analysis for basic variables (summary)

Table 2 summarizes the main results of various diagnostic variables. *Agni (prakriti)* (κ = 0.2; *p* < 0.001), *akriti* (κ = 0.29; *p* < 0.001), *nadi dosha* (κ = 0.18; *p* = 0.040), *agni (vikriti)* (κ = 0.22; *p* < 0.001), and *roga samuha* (κ = −0.30; *p* < 0.001) showed a statistically significant moderate agreement. For all other diagnostic variables, no agreement at all except by chance given random guessing was found.

**Table 2.** Fleiss' Kappa Analysis on Selected Basic Diagnostic Variables

*Fleiss' kappa analysis*						
	*κ*	*Asymptotic standard error*	*Z-value*	p*-Value*	*Lower 95% CI bound*	*Upper 95% CI bound*
*Prashna-pariksha*						
*Agni*	0.2	0.05	3.99	<0.001	0.1	0.29
*Purisha:ama-features*	−0.02	0.08	−0.28	0.778	−0.17	0.13
*Pariksha*						
*Akriti*	0.29	0.04	6.68	<0.001	0.20	0.37
*Nadi dosha*	0.18	0.07	2.52	0.012	0.04	0.32
*Jihva dosha*	−0.01	0.06	−0.18	0.860	−0.13	0.11
*Prakriti*	0.02	0.05	0.52	0.607	−0.07	0.12
*Samprapti*						
*Agni*	0.22	0.05	4.31	<0.001	0.12	0.32
*Kriyakala*	−0.06	0.08	−1	0.472	−0.2	0.09
*Roga samuha*	−0.3	0.08	−4.08	<0.001	−0.45	−0.16

*Agni*, digestive and metabolic capacity; *Akriti*, examination of physique; CI, confidence interval; *Jihva-dosha*, the dominant dosha(s) found in tongue examination; *Kriyakala*, staging of the disease process; *Nadi dosha*, the dominant dosha found in pulse examination; *Pariksha*, diagnostic methods; *Prashna*, question; *Prashna pariksha*, detailed interrogation of the patient for an overall picture of the illness; *Roga samuha*, dosha combination involved in the disease process (“dosha-group” or “disease-group”); *Samprapti*, etiopathogenesis.

CI, confidence interval.

### Inter-rater reliability for the final Ayurvedic diagnosis (viniscita-vyadhi)

In the consensus meeting, 30 patients were diagnosed as *sandhi-gata vata* and 1 patient as *sandhi-gata vata + ama*. Comparing the final diagnosis of the four physicians (P1–P4) with the above consensus diagnosis, the following results were obtained: P1 (κ = 0.37, *p* = 0.010), P2 (Cohen's kappa statistics could not be calculated because all patients were classified as *sandhi-gata vata*, that is, constant; Cohen's kappa cannot be computed when one or both raters give the same rating to all factors), P3 (κ = −0.03, *p* = 0.850), and P4 (κ = 1, *p* < 0.001; P4 = consensus).

Reliability coefficients are in three cases low even though there are high levels of agreement. P1 diagnosed 26 patients as *sandhi-gata vata* and 4 as *sandhi-gata vata + ama*; that is, a total of 3 patients (10%) were “misclassified” as *sandhi-gata vata + ama* instead of *sandhi-gata vata* (compared with consensus agreement). The 10% “misclassification” corresponds to a κ value = 0.37 (fair reliability, *p* = 0.010). The conditional probability for *sandhi-gata vata* is 0.95 in this case. P2 “misclassified” only one patient with final diagnosis *sandhi-gata vata + ama* as *sandhi-gata vata*. The 29 patients with *sandhi-gata vata* were “correctly” classified. Nevertheless, Cohen's algorithm is not able to calculate the corresponding kappa. The Fleiss algorithm resulted in a κ value = 0.02 (*p* = 0.926), a value that is not plausible at all.

Clearly, statistical significance means little in such cases when so much error exists in the results being tested. In contrast to the results of the kappa statistics, the IRA between the four raters and the consensus delivers the following percentages of agreement: P1: 90%; P2: 96.7%; P3: 93.3%; and P4: 100%. This demonstrates again that IRA is a better method when the variable barely varies.

**FIG. 2.**
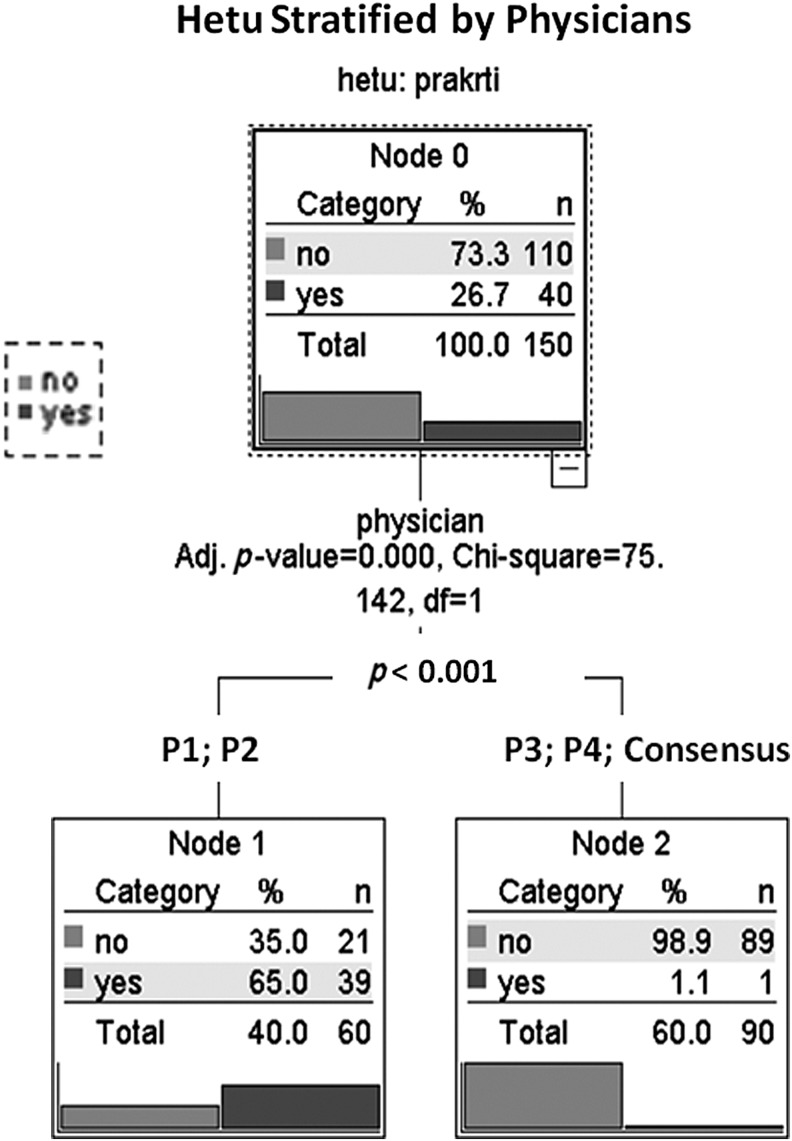
Hetu stratified by physicians. *Hetu*, etiology; *Prakriti*, individual constitution.

Multiple response analyses for the *hetu* component of *samprapati* (most important pathogenic factors for the onset of the disease) reveal similar patterns that can be observed for all four physicians (see Table 3). Even more detailed analyses, however, revealed that there are distinct differences between experts and groups of experts. The example for the individual constitution (*prakriti*) as such an important factor further illuminates this issue. The decision tree (Fig. 2) shows that there are no significant differences between P1 and P2 on one side and P3, P4, and consensus on the other side, but a significant difference (*p* < 0.001) between the two groups P1+P2 and P3+P4+consensus.

**Table 3.** Multiple Response Analysis: Cross-Tabulation Example 1: Hetu

	*Physician*					
	*P1*	*P2*	*P3*	*P4*	*Consensus*	*Total*
*Hetu*^a^						
*Abhigata*						
Count	9	12	19	23	20	83
% within physician	30.0	41.4	63.3	76.7	66.7	
*Ahara*						
Count	20	29	22	27	29	127
% within physician	66.7	100.0	73.3	90.0	96.7	
*Bija dosha*						
Count	0	0	1	1	0	2
% within physician	0.0	0.0	3.3	3.3	0.0	
*Karma*						
Count	1	1	0	0	0	2
% within physician	3.3	3.4	0.0	0.0	0.0	
*Medoroga*						
Count	1	0	0	0	0	1
% within physician	3.3	0.0	0.0	0.0	0.0	
*Nidra*						
Count	1	0	0	0	0	1
% within physician	3.3	0.0	0.0	0.0	0.0	
*Prakrti*						
Count	22	17	0	0	1	40
% within physician	73.3	58.6	0.0	0.0	3.3	
*Vayah*						
Count	1	7	16	28	23	75
% within physician	3.3	24.1	53.3	93.3	76.7	
*Vihara*						
Count	27	29	22	28	29	135
% within physician	90.0	100.0	73.3	93.3	96.7	
*Vyasana*						
Count	1	0	0	0	1	2
% within physician	3.3	0.0	0.0	0.0	3.3	
Total count	30	29	30	30	30	149

Percentages and totals are based on respondents.

^a^ Dichotomy group tabulated at value 1.

*Abhigata*, physical trauma; *Ahara*, diet; *Bija dosha*, congenital factors; *Karma*, work-related activities; *Medoroga*, obesity; *Nidra*, sleep; *Prakriti*, constitution; *Vayah*, age; *Vihara*, behavior; *Vyasana*, addictions.

Analyses as displayed in Table 4 reveal that there is almost perfect inter-rater agreement for most of the *samprapti* categories, including the final Ayurvedic diagnosis (*vinishcita-vyadhi*).

**Table 4.** Multiple Response Analysis: Cross-Tabulation Example 2: Samprapti

	*Physician (%)*						
	*P1*	*P2*	*P3*	*P4*	*Consensus*	*Total (%)*	p*-Value*
*Dosha pradhanya*							
*Vata*	100.0	83.3	100.0	100.0	100.0	96.7	<0.001
*Pitta*		16.7	0.0	0.0	0.0	3.3	
*Dushya*							
*Asthi*	100.0	100.0	100.0	100.0	100.0	100.0	n.s.
*Dushya*							
*Mamsa*	100.0	90.0	100.0	100.0	100.0	100.0	n.s.
*Dushya*							
*Kandara*	96.7	100.0	100.0	100.0	100.0	100.0	n.s.
*Dusya*							
*Snayu*	100.0	100.0	100.0	100.0	100.0	100.0	n.s.
*Agni*							
*Sama*	50.0	60.0	60.0	73.3	70.0	62.7	0.002
*Manda*	13.3	10.0,	26.7	0.0	0.0	10.0	
*Tiksna*	0.0	6.7	0.0	16.7	6.7	6.0	
*Visama*	36.7	23.3	13.3	10.0	23.3	21.3	
*Ama*							
Yes	13.3	16.7	6.7	0.0	0.0	7.4	0.039
No	86.7	83.3	93.3	100.0	100.0	92.6	
*Kriyakala*							
*Vyakti*	0.0	17.2	0.0	3.3	0.0	4.0	0.002
*Bheda*	100.0	82.8	100.0	96.7	100.0	96.0	
*Roga samuha*							
*Pitta-vata vyadhi*	0.0	6.7	0.0	0.0	0.0	1.3	n.s.
*Vata vyadhi*	100.0	93.3	100.0	100.0	100.0	98.7	
*Vinishcita-vyadhi*							
*Sandhi gata vata*	86.7	100.0	96.7	96.7	96.7	95.3	n.s.
*Sandhi gata vata + ama*	13.3	0.0	3.3	3.3	3.3	4.7	

*Dosha pradhanya*, doshic predominance; *Dushya*, affected tissue; *Asthi*, bone tissue and cartilage; *Mamsa*, muscle tissue; *Kandara*, tendons; *Snayu*, nerves and ligaments; *Sama agni*, good digestive capacity; *Manda agni*, poor digestive capacity; *Tikshna agni*, hyperactive digestive capacity; *Vishama agni*, instable digestive capacity; *Ama*, prevalence of toxins; *Kriyakala*, staging of the disease process; *Vyakti*, disease manifestation; *Bheda*, complications; *Roga samuha*, dosha combination involved in the disease process (“dosha-group” or “disease-group”); *Vinishcita-vyadhy*, final diagnosis.

n.s., not significant.

## Discussion

This diagnostic reliability study including 30 patients who were diagnosed by 4 independent Ayurveda physicians is the first of its kind performed within the context of a clinical trial on Ayurveda in a Western setting and one of the very few existing diagnostic trials on Ayurveda at all.^47–49^ Ayurveda treatment was based on Ayurvedic diagnosis only.^5,7^

The importance of rater reliability lies in the fact that it represents the extent to which the data collected in the study are correct representations of the variables measured. While high percentages of agreement for main diagnostic entities and the final Ayurveda diagnosis could be observed, kappa values largely yielded fair-to-poor IRA. This means that the error component is large for that assessment.

One explanation for this effect is that inter- and intrarater reliability are affected by the fineness of discriminations in the data.

The high percentages of agreement for a final Ayurveda diagnosis are not reflected in the corresponding kappa values, showing mostly fair or poor reliability. One consequence could be that kappa statistics is inappropriate at least in such a context. This has been widely observed in other diagnostic studies, for example with similar results in conventional medicine,^52,53^ thus posing a generic challenge not only for diagnostic studies but also for research on traditional systems of medicine.

In general, high levels of disagreement among raters suggest weaknesses in the underlying qualitative notions and the diagnostic methods, including the possibility of poor operational definitions, categories, and perhaps deficiencies in the inter-rater training.^47^

There is another source of concern. Kappa's calculation uses a term called the proportion of chance (or expected) agreement. This is interpreted as the proportion of times raters would agree by chance alone. However, the term is relevant only under the conditions of statistical independence of raters. Since raters are clearly not independent (in this case, all four raters knew that three other raters would also perform their diagnoses), the relevance of this term, and its appropriateness as a correction to actual agreement levels, is questionable. The knowledge that other physicians are also performing the same task may lead to hyperexact performance by the participating physicians. Such a constellation may turn out to be erratic, even more so when three Western raters are aware that a very experienced Indian Ayurveda expert is also performing.

Notably, the group usually followed the Indian expert's opinion as for the consensus rating; interestingly, the Indian expert's ratings are “steadiest” compared with all others' rating patterns (meaning that he gave similar diagnostic patterns most often). It must be considered that the more differentiated different personal qualitative diagnostic systems are, the bigger the differences between raters. By considering the possible errors in decision making on the ratings brought about by experts unintentionally exercising prior clinical experience, influencing their ratings in this study (i.e., preoccupations about certain constitutional features are often observed among Ayurvedists of both Indian and Western provenience), there is a need to counteract this limitation in future studies.^54–56^

Considering the above, there is an urgent need for detailed guidelines to be established for conducting Ayurvedic diagnosis and traditional diagnostic processes in general in clinical trials.

As aforementioned, the diagnostic differences between the Western Ayurveda experts on one hand and the Indian expert on the other hand are obvious. For future diagnostic studies on Ayurveda in such cross-cultural settings/backgrounds, rigorous study methodologies probably also need to include different constitutional perceptions based on different cultural backgrounds, including differences in phenotypic assessments. The same aspects just as well apply to different Ayurveda traditions in India (e.g., Northern vs. Southern traditions) with slightly different diagnostic approaches and interpretations.^19–23,25^

Another aspect that deserves attention in future diagnostic studies on Ayurveda—and clinical trials on whole systems Ayurveda treatment in general—is whether a specific *dosha* might have clearer diagnostic markers than others, both health- and disease specific.

Moreover, questions need to be raised on whether a long-term diagnostic experience (Indian expert) would still generally lead to significant differences in diagnostic results when compared with proportionally rather short-term diagnostic experiences of most Western experts who usually practice Ayurveda as “complementary medicine” alongside conventional medicine or as part of “integrative” approaches, but rarely as “Ayurveda only.”^57^ For the same reason, future IRR studies on Ayurveda should also include a larger number of South Asian Ayurveda physicians formally trained in Ayurveda Universities, with clinical Ayurveda practice being their primary work focus.

The relatively small study sample is in fact the most relevant limitation of this study as of other diagnostic studies^47,48^ as common reliability measure such as Cohen's or Fleiss' kappa is not ideal for measuring reliability in small sample sizes. Using Cohen's or Fleiss' kappa algorithms in rather small sample sizes, as in this study, leads to the peculiar situation of extremely high conditional probability values for some diagnostic entities without being able to calculate corresponding kappa values. There is wide disagreement about the usefulness of kappa statistics to assess rater agreement. Whether a given kappa value implies a good or a bad rating system or diagnostic method depends on what model one assumes about the decision making of raters.^41^ Furthermore, Kappa is influenced by trait prevalence (distribution) and base rates. As a result, kappas are seldom comparable across studies, procedures, or populations, posing a major challenge in the field of diagnostic studies.^58,59^

Another limitation is that all physician raters could have been biased in their assessments by the open-label RCT design; particularly by being aware that all study subjects were participating in a trial on knee osteoarthritis. This aspect should be considered in methodological planning of future diagnostic studies on Ayurveda.

All patients were explicitly instructed to not share any diagnostic information before the commencement of the study and after all rounds of diagnostic assessments on each study day. Compliance regarding this aspect was not documented in written form, thereby marking a minor source of potential bias. However, no single violation of this agreement was reported by study personnel or participants.

Another limiting factor is the fact that two physician raters were also coauthors of this article for editing the Ayurveda background section, creating a potential source of bias. Coauthorship with them was discussed and agreed upon only after the termination of the diagnostic study.

Also, these data raise general questions on the meaning and usefulness of several traditional diagnostic subcomponents of Ayurveda for Ayurveda practice in Western settings, when the diagnostic agreement, particularly between Western raters, is low in many cases (as measured by IRR here), but the ultimate Ayurvedic diagnostic entity (disease classification) is usually agreed upon, and the concrete therapeutic recommendations—and realities—are also in accordance to a large extent.

While the question regarding the importance of the differences in Ayurvedic diagnosis among practitioners is important, it exceeds the research aim of this study and will certainly be a worthwhile topic for further research. Contrary to this, however, it can be argued that one cannot say that diagnostic subcomponents are useless just because they are not assessed as reliable in clinical trial conditions due to possible limitations of statistical methods—the diagnosis in Ayurveda is mainly a matter of perception not deterministic thinking. This can be achieved only if a doctor is educated in this direction, privileging the direct perception of the whole person including all symptoms through one's senses instead of an isolated technical view of a diseased organ or body part, as often done in conventional diagnostic approaches.

## Conclusions

The results address several issues with an important question being whether the use of kappa algorithms to quantify actual levels of agreement between raters is an appropriate technique for analyzing traditional diagnostics, particularly in case of small sample sizes. For the future diagnostic studies with larger sample sizes and a sophisticated methodology, particularly related to statistics, tailored to the specificities of traditional whole systems of medicine are warranted.

## Supplementary Material

Supplemental data

Supplemental data

Supplemental data
