# Supplementary material for: Reliability of Ayurvedic Diagnosis for Knee Osteoarthritis Patients: A Nested Diagnostic Study Within a Randomized Controlled Trial
Source: J Altern Complement Med. 2019 Sep 12;25(9):910–9. doi: 10.1089/acm.2018.0273 (PMC6748397; doi:10.1089/acm.2018.0273)
Supplement: Supplemental data [file Supp_Data1-Fig1.pdf]

Supplementary Data

Supplementary Data S1. Ayurvedic Dosha Concept

Ayurvedic *Dosha* Concept

In Ayurveda, the word *dosha* derived from Sanskrit language implies the three main principles governing the physiological activities in the human body. The three *doshas* are *vata, pitta,* and *kapha.*[S1,S2] In their normal states, the three *doshas* contribute toward maintaining physiological balance, whereas in their abnormal states the three *doshas* become pathologic, thus resulting in various types of illnesses.[S3,S4] According to Ayurvedic theory, the *doshas* are always susceptible to disequilibrium, and have a tendency to vitiate other bodily tissues and functions.

The theory of the *doshas* forms the fundamental principle in Ayurvedic thinking. Without understanding the concept of the three *doshas*, it is very unlikely to appreciate any other principles of Ayurveda. As such, the three *doshas* are referred to as *tristhuna* in Sanskrit, meaning ''the three pillars,'' describing the role of the three *doshas* in the human body as ''pillars holding a building.''[S5]

Modern thinking, in particular science, largely accepts materialistically perceptible entities and as such tends to interpret Ayurvedic concepts by making references to biomechanical aspects of human functions. As a result, practitioners of Ayurveda also try to understand and interpret *doshas* to material equivalents in the body such as blood, enzymes, tissues, and hormones. However, according to the traditions of Ayurveda *doshas* are not material entities. As energy can usually not be perceived through the senses but is inferred through its actions, the *doshas* are also imperceptible through the senses but are inferable through their properties and functions.

According to Ayurvedic theory, the body's interior milieu is the most important factor behind every activity of the body and is classified on the basis of its perceptible properties; for example, roughness, oiliness, heat, cold, viscosity, acidity, alkalinity, sweetness, lightness, or heaviness. Ayurveda classifies those properties in three large categories, the three *doshas* as *vata*-related, *pitta*-related, and *kapha*-related milieus. All bodily factors act according to the milieu available to them. Thus, *doshas* are not to be understood as some particular matter or substance but as property-driven compound phenomena producing certain patterns of milieus atmospheres in which certain patterns of physiologic or pathophysiologic responses are generated.

Vata dosha

The word *vata* originates from the Sanskrit root ''*va*'' denoting movement. Thus, *vata* may be understood as the kinetic principle responsible for all types of movements in the body. *Vata* is characterized by lightness, dryness, roughness, nonsliminess, coldness, mobility, and subtleness. Thus, it may produce and maintain these qualities in the body, and gives rise to a milieu favorable to *vata*-related activities. When provoked, *vata* may abnormally increase these qualities resulting in an atmosphere in which certain activities become abnormal, and thus causing *vata*-related diseases. Through its specific qualities, *vata* performs respiration, muscular movements, circulation, excretion, sensory impulses, speech, fetal developments, and everything else that requires any kind of movement.

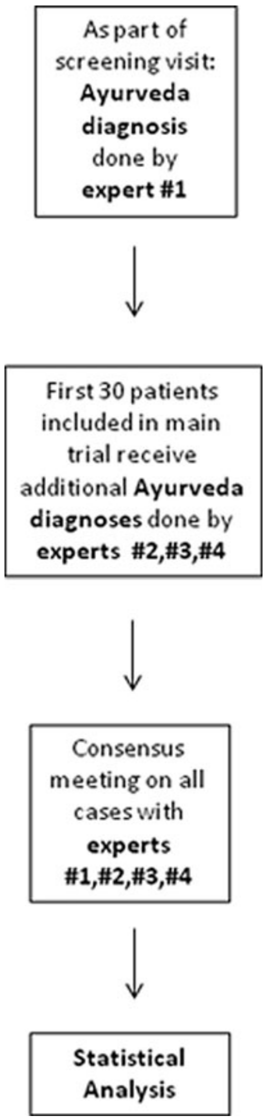

SUPPLEMENTARY FIG. S1. Study design

Pitta dosha

Pitta is the thermal principle, the term originating from the Sanskrit root ''*tapa*'' denoting ''heat.'' Hence, *pitta* is responsible for heat, energy, and light-related activities. *Pitta is* characterized by slight oiliness or moisture, heat, liquidity, sourness, pungency and sharpness. Thus, in its normal state *pitta* produces and maintains these qualities in the body. When provoked, *pitta* may cause an abnormal increase in these qualities, which may result in diseases. *Pitta*

is responsible for digestion, metabolism, energy production, heat maintenance, visual function, valor, anger, hunger, thirst, and intelligence. Its activities are most prominent in the umbilical region, small intestines, and the lower parts of the stomach. These regions are known as the seats of *pitta.*

Kapha dosha

Kapha is the hydroic principle derived from its Sanskrit root ''*ka*'' denoting ''water.'' *Shleshma,* a synonym for *kapha,* means ''to embrace'' referring to Kapha's function of ''holding tissues together.'' *Kapha* is characterized by oiliness, coldness, heaviness, sweetness, stability, sliminess or being sticky (causing adherence) and softness. Thus, in its normal state *kapha* produces and maintains these aforementioned qualities in the body, and when provoked it may cause an abnormal increase in these qualities, therefore producing diseases. *Kapha* is responsible for anabolism, strength, potency, stability, lubrication, nourishment, tolerance, and contentment. Its activities are most prominent in chest, throat, head, joints, and upper parts of the stomach. These regions are known as seats of *kapha.*

Supplementary References

S1. Sharma PV (transl.). Carakasamhita. Text with English translation. 7th Edition. Sūtra Sthāna: 1.57. Varanasi, India: Chaukhambha Orientalia, 2005.

S2. Srikantha Murthy KR. Vaghbata Astanga Hrdayam. Sūtra Sthāna: 1.6. Varanasi, India: Chaukhambha Orientalia, 2007.

S3. Sharma PV (transl.). Carakasamhita. Text with English translation. 7th Edition. Vimana-sthana: 1.5. Varanasi, India: Chaukhambha Orientalia, 2005.

S4. Sharma PV (transl.). Carakasamhita. Text with English translation. 7th Edition. Sūtra Sthāna: 20.9. Varanasi, India: Chaukhambha Orientalia, 2005.

S5. Sharma PV (transl.). Sushruta Samhita. English translation. Reprint 2010. Sutrasthana (Su.): 21.4. Varanasi, India: Chaukhambha Visvabharati, 2010.
