# Supplementary material for: Reliability of Ayurvedic Diagnosis for Knee Osteoarthritis Patients: A Nested Diagnostic Study Within a Randomized Controlled Trial
Source: J Altern Complement Med. 2019 Sep 12;25(9):910–9. doi: 10.1089/acm.2018.0273 (PMC6748397; doi:10.1089/acm.2018.0273)

**Supplementary Data**

**
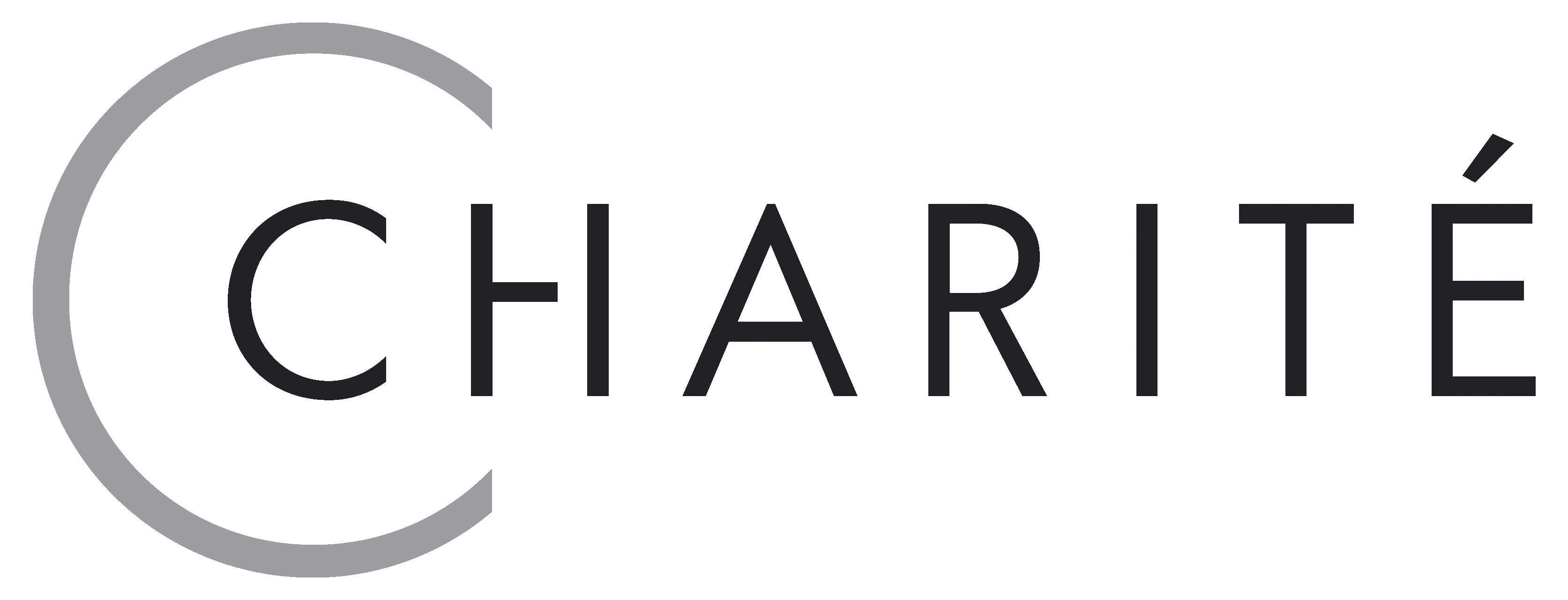
**

Charité – Universitätsmedizin Berlin

Institut für Sozialmedizin, Epidemiologie und Gesundheitsökonomie

Direktor: Prof. Dr. Stefan N. Willich, MPH, MBA

und Gesundheitsökonomie

**CARAKA-Study / CARAKA-Studie**

***PARIKSA Konsensus-Bogen / Consensus-Sheet***

| Patientencode:  ëûëûëû | **Prüfarzt:**  **Prüfzentrum:**  (BLOCKBUCHSTABEN) | **Datum:** ëûëû.ëûëû.ëûëû | |
| --- | --- | --- | --- |
|  |  | **laufende**  **Screening-**  **Nummer:** | ëûëûëû |


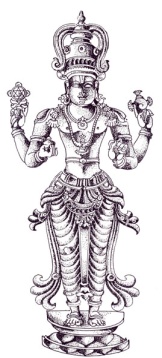


**Supplementary Data S2. Diagnostic_Assessment_Form**

| 1. ***samprāpti (Pathogenesis / Pathogenese)*** | | | | | | |
| --- | --- | --- | --- | --- | --- | --- |
| 1. **hetu** | | |  | | | |
| 1. **doṣa prādhānya** | | | ❒ vāta ❒ pitta ❒ kapha | | | |
| 1. **dūṣya** | | | ❒ asthi ❒ māṃsa ❒ kaṇḍharā ❒ snāyu | | | |
| 1. **agni** | | | ❒ sāma ❒ manda ❒ tīkṣna ❒ viśama | | | |
| 1. **āma** | | | ❒ yes / ja ❒ no / nein | | | |
| 1. **krīyākāla** | | | ❒ saṃcaya ❒ prakopa ❒ prasara ❒ sth. saṃśraya ❒ vyakti ❒ bheda | | | |
| 1. **roga samūha** (group of diseases / übergeordnete Gruppe von Erkrankungen) | | |  | | | |
|  | | |  | | | |
| **viniścita-vyadhi** (ayurvedic diagnosis / ayurvedische Diagnose) | | |  | | | |
|  | | |  | | | |
| 1. ***cikitsā / Advised Treatment*** | | | | | | |
| ***auṣadhi / dietary supplements / Nahrungsergänzungen*** | | | | | | |
| **auṣadhi yoga/ Name** | **mātrā / Dosage / Dosis** | | | | | **anupāna / Mode of administration / Verabreichung** |
| ❒ **Yogarāja-Guggulu** | , g ;  -  -  (daily/tgl.) | | | | | ❒ warm milk/ ❒ hot water ❒ ghī  warme Milch heißes Wasser |
| ❒ **Kaiśora-Guggulu** | , g ;  -  -  (daily/tgl.) | | | | | ❒ warm milk/ ❒ hot water ❒ ghī  warme Milch heißes Wasser |
| ❒ **Aśvagandhā Vaṭi** | , g ;  -  -  (daily/tgl.) | | | | | ❒ warm milk/ ❒ hot water ❒ ghī  warme Milch heißes Wasser |
| ❒ **Pippalī Vaṭi** | , g ;  -  -  (daily/tgl.) | | | | | ❒ warm milk/ ❒ hot water ❒ ghī  warme Milch heißes Wasser |
| ❒ **Tagara Vaṭi** | , g ;  -  -  (daily/tgl.) | | | | | ❒ warm milk/ ❒ hot water ❒ ghī  warme Milch heißes Wasser |
| ❒ **Eraṇḍamūlakvātha** |  mL ;  -  -  (daily/tgl.) | | | | | ❒ warm milk/ ❒ hot water ❒ ghī  warme Milch heißes Wasser |
| ❒ **Balāmūlakvātha** |  mL ;  -  -  (daily/tgl.) | | | | | ❒ warm milk/ ❒ hot water ❒ ghī  warme Milch heißes Wasser |
| ❒ **Manjiṣṭhadikvātha** |  mL ;  -  -  (daily/tgl.) | | | | | ❒ warm milk/ ❒ hot water ❒ ghī  warme Milch heißes Wasser |
| ❒ **Punarnavādikvātha** |  mL ;  -  -  (daily/tgl.) | | | | | ❒ warm milk/ ❒ hot water ❒ ghī  warme Milch heißes Wasser |
| ❒ **Mahānārāyaṇatailam** |  mL ;  -  -  (daily/tgl.) | | | | | ❒ warm milk/ ❒ hot water ❒ ghī  warme Milch heißes Wasser |
| ❒ **Cyavanaprāśāvaleha** |  TS/EL ;  -  -  (daily/tgl.) | | | | | ❒ warm milk/ ❒ hot water ❒ ghī  warme Milch heißes Wasser |
| ❒ **__________________** |   __ ;  -  -  (___/___) | | | | | ❒   _________________________ |
| ❒ **__________________** |   __ ;  -  -  (___/___) | | | | | ❒ _________________________ |
| ❒ **__________________** |   __ ;  -  -  (___/___) | | | | | ❒ _________________________ |
|  |  | | | | |  |
| ***bāhyopacara / local applications / lokale Anwendungen*** | | | | | | |
| **bāhyopacāra /****Name** | | | | **āvṛtti / Frequency of application / Frequenz** | | |
| ❒ **Guggulu-lepa** | | | |  / week / Woche | | |
| ❒ **Nimba-patra-upanāha** | | | |  × week / Woche | | |
| ❒ **Nirguṇḍi-patra-upanāha** | | | |  × week / Woche | | |
| ❒ **__________________________** | | | |  × week / Woche | | |
|  | | | |  | | |
| ***śodhana / purification procedures / Reinigungsprozeduren*** | | | | | | |
| **karma / Name** | | **āvṛtti, mātrā / Frequency and Dosage of application /  Frequenz und Dosis** | | | | |
| ❒ **mṛdu-virecana** | | ❒eraṇḍa-sneha;  mL  ❒harītakī cūrna ❒dīnadāyal-cūrna ❒ bitter salts;  g | | | | |
| ❒ **tila-tailam-mātra-svābasti** | |  / week / Woche;  mL | | | | |
| ❒ **_____________________** | |  | | | | |
|  | |  | | | | |
| ***abhyaṇga, svedana / manual therapies and sudation / Manuelle Therapie, Sudation*** | | | | | | |
| **āvṛtti / frequency / Frequenz** | | | | | **āvṛtti / Frequency / Frequenz** | |
| ❒  **mahānārāyaṇatailam sarvangābhyaṇga** | | | | |  in 12 weeks / Wochen (max. 15) | |
| ❒  **dhānvaṇtaratailam sarvangābhyaṇga** | | | | |  in 12 weeks / Wochen (max. 15) | |
| ❒  **yava-sarvanga-udvartana** | | | | |  in 12 weeks / Wochen (max. 15) | |
| ❒  **mahānārāyanatailam jānu-abhyanga** | | | | |  in 12 weeks / Wochen (max. 15) | |
| ❒  **dhānvantaratailam jānu- abhyanga** | | | | |  in 12 weeks / Wochen (max. 15) | |
| ❒  **mahānārāyanatailam jānu-svābhyanga** | | | | |  per week / pro Woche | |
| ❒  **dhānvantaratailam jānu-svābhyanga** | | | | |  per week / pro Woche | |
| ❒  **svedana** | | | | |  in 12 weeks / Wochen (max. 15) | |
| ❒  **___________________________________** | | | | |  | |
|  | | | | |  |  |
| ***vyāyāma-yoga / supportive Yoga / unterstützende Yogaübungen*** | | | | | | |
| ❒ **yes / ja** ❒ **no / nein** | | | | | | |
|  | | | | | | |
| ***āhāra / dietary advice / Ernährungsempfehlungen*** | | | | | | |
| ❒ **yes / ja** ❒ **no / nein** | | | | | | |
|  | | | | | | |
| ***vihāra / lifestyle advice / Lebensstilempfehlungen*** | | | | | | |
| ❒ **yes / ja** ❒ **no / nein** | | | | | | |


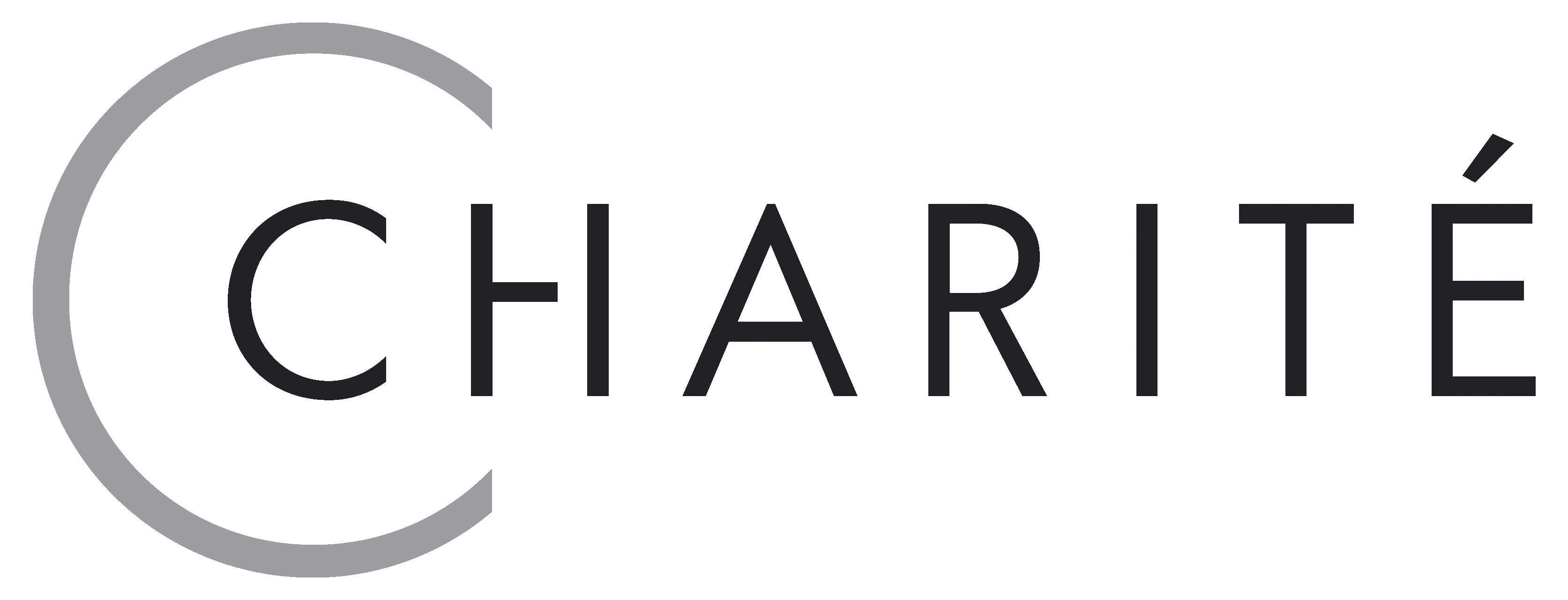

Supplement: Supplemental data [file Supp_Data2.doc]
