# Supplementary material for: Reliability of Ayurvedic Diagnosis for Knee Osteoarthritis Patients: A Nested Diagnostic Study Within a Randomized Controlled Trial
Source: J Altern Complement Med. 2019 Sep 12;25(9):910–9. doi: 10.1089/acm.2018.0273 (PMC6748397; doi:10.1089/acm.2018.0273)
Supplement: Supplemental data [file Supp_Data3.doc]

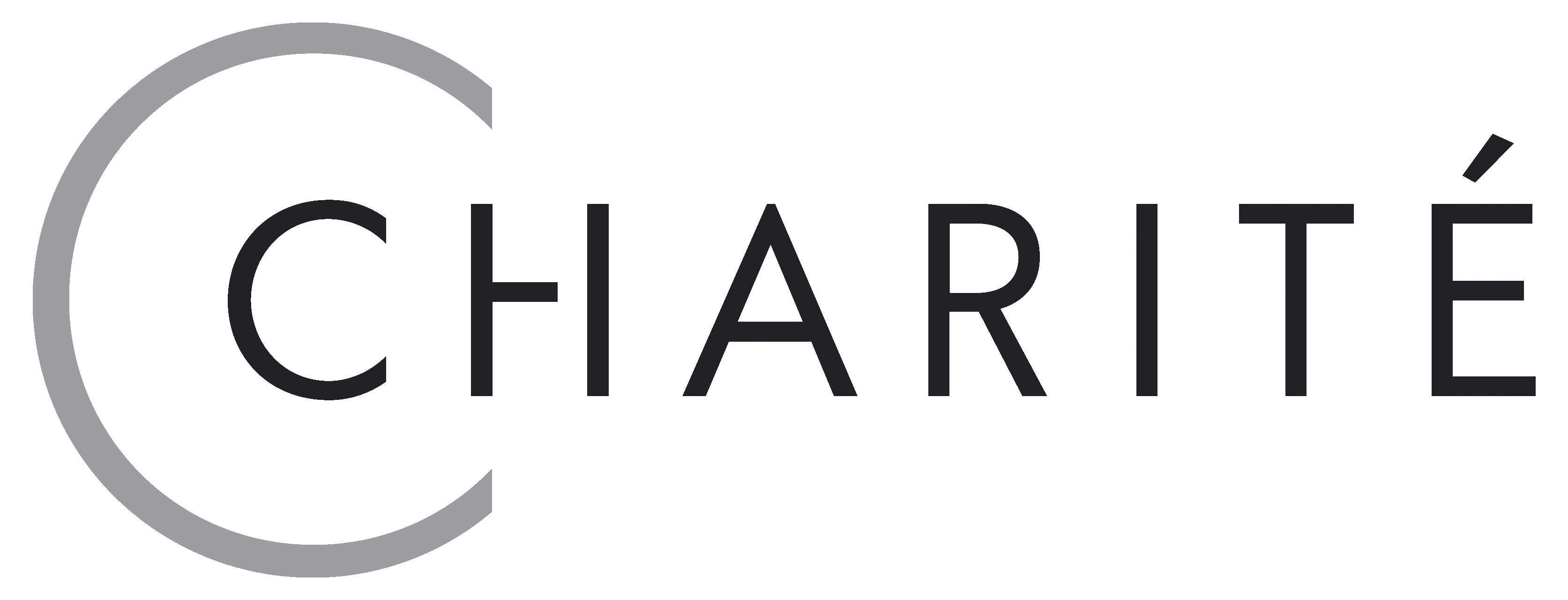


Charité – Universitätsmedizin Berlin

Institut für Sozialmedizin, Epidemiologie und Gesundheitsökonomie

Direktor: Prof. Dr. Stefan N. Willich, MPH, MBA

und Gesundheitsökonomie

**CARAKA-Study / CARAKA-Studie: PARIKSA**

***prathama* *āturavṛtta /* Āyurveda-Visit 1 / -Visite 1**

| Patientencode:  ëûëûëû | **Prüfarzt:**  **Prüfzentrum:**  (BLOCKBUCHSTABEN) | **Datum:** ëûëû.ëûëû.ëûëû | |
| --- | --- | --- | --- |
|  |  | **laufende**  **Screening-**  **Nummer:** | ëûëûëû |


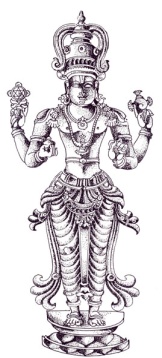


Supplementary Data S3. Diagnostic_Consensus_Form

| 1. ***praśna-parīkṣā* (Patient History / Anamnese)** | | | | | | | | |
| --- | --- | --- | --- | --- | --- | --- | --- | --- |
| 1. ***sāṃpratvyādhivṛtta*  (present complaints and symptoms, history, onset and progress of present illness / Aktuelle Beschwerdesymptomatik, Geschichte, Beginn und Verlauf der aktuellen Erkrankung)** | | | | | | | | |
| 1. ***pūrvavyādhivṛtta*  (relevant other past illnesses and surgery / relevante andere Erkrankungen und Operationen)** | | | | | | | | |
| 1. ***kulavṛtta* (disease-relevant family history / krankheitsrelevante Familenanamnese)** | | | | | | | | |
| 1. ***saṃbaddhavaiyaktiktajīvanavṛtta*  (relevant life incidents related to present illness / relevante Ereignisse im Leben, die mit der Erkrankung in Zusammenhang stehen)** | | | | | | | | |
| 1. **agni** | ❒ sāma ❒ maṇḍa ❒ tīkṣna ❒ viśama | | | | | | | |
| 1. **koṣṭha**   **Defecation**  **Stuhlgang** | ❒ krūra ❒ mṛdu ❒ madhya | | | | | | | |
| 1. **purīṣa**   **Stool**  **Stuhl** | āvṛtti (frequency / Frequenz): ëûëû / day / Tag ❒ irregular / unregelmäßig | | | | | | | |
|  | svarūpa (consistency / Konsistenz): | | | | | | | |
|  | varṇa (color / Farbe): | | | | | | | |
|  | gandha (smell / Geruch): | | | | | | | |
|  | āma-features / āma-Zeichen : ❒ no / nein ❒ yes / ja | | | | | | | |
|  | ❒ if yes, specify / wenn ja, welche: | | | | | | | |
| 1. **Mūtra**   **Micturation / Urine**  **Miktion / Urin** | āvṛtti (frequency / Frequenz): ëûëû / day / Tag ❒ irregular / unregelmäßig | | | | | | | |
|  | svarūpa (clarity / Klarheit): | | | | | | | |
|  | varṇa (color / Farbe): | | | | | | | |
|  | gandha (smell / Geruch): | | | | | | | |
| 1. **Svapna**   **Dreams**  **Träume** | Specific dreams / Besondere Träume? ❒ no / nein ❒ yes / ja | | | | | | | |
|  | If yes, specify / wenn ja, welche: | | | | | | | |
|  | āvṛtti (frequency / Frequenz): ëûëû / week / Woche ❒ irregular / unregelmäßig | | | | | | | |
| 1. **Nidrā**   **Sleep**  **Schlaf** | Duration in hours / Dauer: ëûëû h / night / Nacht | | | | | | | |
|  | Number of Interruptions / Unterbrechungen: ëûëû / night / Nacht | | | | | | | |
|  | Quality / Qualität: ❒ deep / tief ❒ medium / mittel ❒ light / leicht | | | | | | | |
|  | Feeling after waking up / ❒ energetic / ❒ fresh / ❒ dull / ❒ tired / Gefühl nach dem Aufstehen: energetisch frisch matt müde | | | | | | | |
|  | Daysleep / Tagesschlaf: ❒ yes / ja ❒ no / nein | | | | | | | |
|  | Onset of sleeping disorders if present / Beginn der Schlafstörung, falls vorhanden:  ❒ before onset of joint pain /vor Auftreten der Gelenkbeschwerden  ❒ after onset of joint pain /nach Auftreten der Gelenkbeschwerden | | | | | | | |
| 1. **Karma**   **Work**  **Arbeit** | Type / Art: | | | | | | | |
|  | Quality / Qualität: ❒ labour / manuell ❒ sitting / sitzend ❒ other / sonst.  | | | | | | | |
|  | work hours / Arbeitsstunden: ëûëû / day / Tag | | | | | | | |
|  | Satisfaction / Zufriedenheit: ❒ very much / ❒ medium / ❒ rather not / ❒ not at all /  sehr mäßig eher nicht gar nicht | | | | | | | |
| 1. **abhiruci /**   **Hobbies** | specify / nähere Angaben: | | | | | | | |
| 1. **vyasana**   **Addictions**  **Abhängig-**  **keiten** | ❒ Alcohol / Alkohol ❒ Nicotine / Nikotin ❒ Caffeine / Koffein  ❒ other / andere  if yes, specify / wenn ja, welche: | | | | | | | |
| 1. **ārtava-pravṛtti**   **Menstruation** | Duration of menstrual cycle / Dauer des Menstruationszyklus: ëûëû days / Tage | | | | | | | |
|  | Type of bleeding / Art der Blutung: ❒ strong/stark ❒ medium/mäßig ❒ light/leicht | | | | | | | |
|  | Associated symptoms / Assoziierte Symptome: | | | | | | | |
| 1. **ārtava-nivṛtti**   **Menopause** | Start of menopause / Beginn der Menopause: ëûëû age / Alter | | | | | | | |
|  | Specific symptoms / Besondere Symptome: | | | | | | | |
|  |  | | | | | | | |
| 1. ***parīkṣā* (General Clinical Examination / Allgemeine klinische Untersuchung)** | | | | | | | | |
| 1. **ākṛti** | ❒ emaciated ❒lean  ❒ medium ❒ stout ❒ obese ❒ over-obese  kachektisch schlank mittel kräftig adipös sehr adipös | | | | | | | |
| 1. **nāḍī** | doṣa: ❒ vāta ❒ pitta ❒ kapha | | | | | | | |
|  | pūrnatā: ❒ pūrna ❒ śaitilya ❒ madhya | | | | | | | |
|  | gati: ❒ druta ❒ madhya ❒ maṇḍa | | | | | | | |
|  | tāla: ❒ sāma ❒ visama | | | | | | | |
|  | sparśa: ❒ kaṭhina ❒ madhya ❒ mṛdu ❒ uṣṇa ❒ śīta | | | | | | | |
|  | āma: ❒ yes / ja ❒ no / nein ❒ not assessable / nicht beurteilbar | | | | | | | |
| 1. **jihvā** | doṣa: ❒ vāta ❒ pitta ❒ kapha | | | | | | | |
|  | varṇa: | | | | | | | |
|  | āma: ❒ yes / ja ❒ no / nein | | | | | | | |
|  | Surface (descriptive) / Oberfläche (deskriptiv): | | | | | | | |
| 1. **ātura-bala-pramāṇa** | prakṛti: ❒ V ❒ P ❒ K ❒ VP ❒ VK ❒ PK ❒ VPK | | | | | | | |
|  | prakṛti: ❒ pravara  ❒ madhya ❒ avara | | | | | | | |
|  | sāra: ❒ pravara   ❒ madhya  ❒ avara | | | | | | | |
|  | pramāna: ❒ pravara  ❒ madhya  ❒ avara | | | | | | | |
|  | sātmya: ❒ pravara  ❒ madhya  ❒ avara | | | | | | | |
|  | sattva: ❒ pravara  ❒ madhya  ❒ avara | | | | | | | |
|  | ahāra-śakti: ❒ pravara  ❒ madhya  ❒ avara | | | | | | | |
|  | vyāyāma-śakti : ❒ pravara  ❒ madhya  ❒ avara | | | | | | | |
|  | vayas: ❒ pravara  ❒ madhya  ❒ avara | | | | | | | |
|  | viniścita-bala: ❒ pravara  ❒ madhya  ❒ avara | | | | | | | |
|  |  | | | | | | | |
| 1. ***jānu-parīkṣā (Clinical Examination of the Knee / Klinische Untersuchung des Knies)*** | | | | | | | | |
| 1. **sandhi-śūla** (joint pain / Gelenkschmerz) | | | | | | | ❒ yes / ja ❒ no / nein | |
| 1. **asthi-śūla / asthi-bheda** (bone pain / Knochenschmerzen) | | | | | | | ❒ yes / ja ❒ no / nein | |
| 1. **asthi- śūṇyatā** (feeling of emptiness in the bones / Gefühl von Leere in den Knochen) | | | | | | | ❒ yes / ja ❒ no / nein | |
| 1. **sandhi-śūṇyatā** (feeling of emptiness in joint / Gefühl von Leere im Kniegelenk) | | | | | | | ❒ yes / ja ❒ no / nein | |
| 1. **prasāraṇākuñcanayoḥ-pravṛttisavedanaṃ** (painful (movements / schmerzhafte Bewegungen) | | | | | | | ❒ yes / ja ❒ no / nein | |
| 1. **apravṛtti** (immobility / Immobilität) | | | | | | | ❒ yes / ja ❒ no / nein | |
| 1. **vātapūrṇadṛtisparśa** (feeling of joint ballooning / ballonierendes Gelenkgefühl) | | | | | | | ❒ yes / ja ❒ no / nein | |
| 1. **sandhiraukṣya** (feeling of joint dryness / Gefühl von Gelenktrockenheit) | | | | | | | ❒ yes / ja ❒ no / nein | |
| 1. **māṃsa-bala-kṣaya** (diminished muscle power / verminderte Muskelkraft) | | | | | | | ❒ yes / ja ❒ no / nein | |
| 1. **sandhi-stabdhatā** (joint stiffness / Gelenksteifigkeit) | | | | | | | ❒ yes / ja ❒ no / nein | |
| 1. **sandhi-śaithilya** (joint loosening / Gelenklockerung) | | | | | | | ❒ yes / ja ❒ no / nein | |
| 1. **sandhi-śotha** (joint swelling / Gelenkschwellung) | | | | | | | ❒ yes / ja ❒ no / nein | |
| 1. **sandhi-sphūṭana** (crepitation / Krepitation) | | | | | | | ❒ yes / ja ❒ no / nein | |
|  | | | | | | |  | |
| 1. ***samprāpti (Pathogenesis / Pathogenese)*** | | | | | | | | |
| 1. **hetu** | | | |  | | | | |
| 1. **doṣa prādhānya** | | | | ❒ vāta ❒ pitta ❒ kapha | | | | |
| 1. **dūṣya** | | | | ❒ asthi ❒ māṃsa ❒ kaṇḍharā ❒ snāyu | | | | |
| 1. **agni** | | | | ❒ sāma ❒ manda ❒ tīkṣna ❒ viśama | | | | |
| 1. **āma** | | | | ❒ yes / ja ❒ no / nein | | | | |
| 1. **krīyākāla** | | | | ❒ saṃcaya ❒ prakopa ❒ prasara ❒ sth. saṃśraya ❒ vyakti ❒ bheda | | | | |
| 1. **roga samūha** (group of diseases / Gruppe von Erkrankungen) | | | |  | | | | |
|  | | | |  | | | | |
| **viniścita-vyadhi** (ayurvedic diagnosis / ayurvedische Diagnose) | | | |  | | | | |
|  | | | |  | | | | |
| 1. ***cikitsā / Advised Treatment*** | | | | | | | | |
| ***auṣadhi / dietary supplements / Nahrungsergänzungen*** | | | | | | | | |
| **auṣadhi yoga/ Name** | | **mātrā / Dosage / Dosis** | | | | | | **anupāna / Mode of administration / Verabreichung** |
| ❒ **Yogarāja-Guggulu** | | , g ;  -  -  (daily/tgl.) | | | | | | ❒ warm milk/ ❒ hot water ❒ ghī  warme Milch heißes Wasser |
| ❒ **Kaiśora-Guggulu** | | , g ;  -  -  (daily/tgl.) | | | | | | ❒ warm milk/ ❒ hot water ❒ ghī  warme Milch heißes Wasser |
| ❒ **Aśvagandhā Vaṭi** | | , g ;  -  -  (daily/tgl.) | | | | | | ❒ warm milk/ ❒ hot water ❒ ghī  warme Milch heißes Wasser |
| ❒ **Pippalī Vaṭi** | | , g ;  -  -  (daily/tgl.) | | | | | | ❒ warm milk/ ❒ hot water ❒ ghī  warme Milch heißes Wasser |
| ❒ **Tagara Vaṭi** | | , g ;  -  -  (daily/tgl.) | | | | | | ❒ warm milk/ ❒ hot water ❒ ghī  warme Milch heißes Wasser |
| ❒ **Eraṇḍamūlakvātha** | |  mL ;  -  -  (daily/tgl.) | | | | | | ❒ warm milk/ ❒ hot water ❒ ghī  warme Milch heißes Wasser |
| ❒ **Balāmūlakvātha** | |  mL ;  -  -  (daily/tgl.) | | | | | | ❒ warm milk/ ❒ hot water ❒ ghī  warme Milch heißes Wasser |
| ❒ **Manjiṣṭhadikvātha** | |  mL ;  -  -  (daily/tgl.) | | | | | | ❒ warm milk/ ❒ hot water ❒ ghī  warme Milch heißes Wasser |
| ❒ **Punarnavādikvātha** | |  mL ;  -  -  (daily/tgl.) | | | | | | ❒ warm milk/ ❒ hot water ❒ ghī  warme Milch heißes Wasser |
| ❒ **Mahānārāyaṇatailam** | |  mL ;  -  -  (daily/tgl.) | | | | | | ❒ warm milk/ ❒ hot water ❒ ghī  warme Milch heißes Wasser |
| ❒ **Cyavanaprāśāvaleha** | |  TS/EL ;  -  -  (daily/tgl.) | | | | | | ❒ warm milk/ ❒ hot water ❒ ghī  warme Milch heißes Wasser |
| ❒ **__________________** | |   __ ;  -  -  (___/___) | | | | | | ❒   _________________________ |
| ❒ **__________________** | |   __ ;  -  -  (___/___) | | | | | | ❒ _________________________ |
| ❒ **__________________** | |   __ ;  -  -  (___/___) | | | | | | ❒ _________________________ |
|  | |  | | | | | |  |
| ***bāhyopacara / local applications / lokale Anwendungen*** | | | | | | | | |
| **bāhyopacāra /****Name** | | | | | **āvṛtti / Frequency of application / Frequenz** | | | |
| ❒ **Guggulu-lepa** | | | | |  / week / Woche | | | |
| ❒ **Nimba-patra-upanāha** | | | | |  × week / Woche | | | |
| ❒ **Nirguṇḍi-patra-upanāha** | | | | |  × week / Woche | | | |
| ❒ **__________________________** | | | | |  × week / Woche | | | |
|  | | | | |  | | | |
| ***śodhana / purification procedures / Reinigungsprozeduren*** | | | | | | | | |
| **karma / Name** | | | **āvṛtti, mātrā / Frequency and Dosage of application /  Frequenz und Dosis** | | | | | |
| ❒ **mṛdu-virecana** | | | ❒eraṇḍa-sneha;  mL  ❒harītakī cūrna ❒dīnadāyal-cūrna ❒ bitter salts;  g | | | | | |
| ❒ **tila-tailam-mātra-svābasti** | | |  / week / Woche;  mL | | | | | |
| ❒ **_____________________** | | |  | | | | | |
|  | | |  | | | | | |
| ***abhyaṇga, svedana / manual therapies and sudation / Manuelle Therapie, Sudation*** | | | | | | | | |
| **āvṛtti / frequency / Frequenz** | | | | | | **āvṛtti / Frequency / Frequenz** | | |
| ❒  **mahānārāyaṇatailam sarvangābhyaṇga** | | | | | |  in 12 weeks / Wochen (max. 15) | | |
| ❒  **dhānvaṇtaratailam sarvangābhyaṇga** | | | | | |  in 12 weeks / Wochen (max. 15) | | |
| ❒  **yava-sarvanga-udvartana** | | | | | |  in 12 weeks / Wochen (max. 15) | | |
| ❒  **mahānārāyanatailam jānu-abhyanga** | | | | | |  in 12 weeks / Wochen (max. 15) | | |
| ❒  **dhānvantaratailam jānu- abhyanga** | | | | | |  in 12 weeks / Wochen (max. 15) | | |
| ❒  **mahānārāyanatailam jānu-svābhyanga** | | | | | |  per week / pro Woche | | |
| ❒  **dhānvantaratailam jānu-svābhyanga** | | | | | |  per week / pro Woche | | |
| ❒  **svedana** | | | | | |  in 12 weeks / Wochen (max. 15) | | |
| ❒  **___________________________________** | | | | | |  | | |
| ❒  **___________________________________** | | | | | |  | | |
|  | | | | | |  | |  |
| ***vyāyāma-yoga / supportive Yoga / unterstützende Yogaübungen*** | | | | | | | | |
| ❒ **yes / ja** ❒ **no / nein** | | | | | | | | |
|  | | | | | | | | |
| ***āhāra / dietary advice / Ernährungsempfehlungen*** | | | | | | | | |
| ❒ **yes / ja** ❒ **no / nein** | | | | | | | | |
|  | | | | | | | | |
| ***vihāra / lifestyle advice / Lebensstilempfehlungen*** | | | | | | | | |
| ❒ **yes / ja** ❒ **no / nein** | | | | | | | | |

| Datum /  Date: ëûëû.ëûëû.ëûëû | Unterschrift des Arztes /  Signature of the Physician: ____________________________________ |
| --- | --- |
